# Authentication of* Coffea arabica* according to Triacylglycerol Stereospecific Composition

**DOI:** 10.1155/2016/7482620

**Published:** 2016-07-28

**Authors:** L. Cossignani, D. Montesano, M. S. Simonetti, F. Blasi

**Affiliations:** Department of Pharmaceutical Sciences, Section of Food Science and Nutrition, University of Perugia, Via San Costanzo, 06126 Perugia, Italy

## Abstract

Stereospecific analysis is an important tool for the characterization of lipid fraction of food products. In the present research, an approach to characterize* arabica* and* robusta* varieties by structural analysis of the triacylglycerol (TAG) fraction is reported. The lipids were Soxhlet extracted from ground roasted coffee beans with petroleum ether, and the fatty acids (FA) were determined as their corresponding methyl esters. The results of a chemical-enzymatic-chromatographic method were elaborated by a chemometric procedure, Linear Discriminant Analysis (LDA). According to the total and intrapositional FA composition of TAG fraction, the obtained results were able to characterize roasted pure coffee samples and coffee mixtures with 10%* robusta* coffee added to* arabica* coffee. Totally correct classified samples were obtained when the TAG stereospecific results of the considered coffee mixture (90 : 10* arabica*/*robusta*) were elaborated by LDA procedure.

## 1. Introduction

Coffee is one of the most popular drinks across the world. From the commercial point of view, only* Coffea arabica* and* C. canephora *var.* robusta* (commonly known as* arabica* and* robusta*, resp.) represent the two most relevant and widely cultivated species [1]. Most commercially available coffee mixtures are in fact obtained from* arabica* and* robusta* blends. These typologies differ not only in relation to their botanical, chemical, and organoleptic characteristics, but also in terms of commercial value; in fact,* arabica* is more expensive due to the high quality [2, 3]. Green coffee beans of the* arabica* and* robusta* varieties can be distinguished by their size, shape, and colour, but the roasting process eliminates these macroscopic aspects [4].

Since the main fraud involving coffee is the undeclared addition of* robusta* to* arabica *variety, there are important economical reasons to demand warranties on the authenticity of coffee species, even if the identification and the quantification of* arabica* in roasted and minced coffee blends are very challenging [5].

Methods identifying markers that can distinguish between the two varieties have been studied for a long time and in the field of chemical analysis several approaches have been applied by considering single compounds or class of compounds, such as caffeine [6], amino acid enantiomers [7], chlorogenic acids and lactones [6, 8], cinnamic acids [9], sugars and other hydrosoluble compounds [10], metals [11], and betaines [12].

Several studies have reported the discrimination between* arabica* and* robusta* coffee throughout lipid components, namely, sterols [13, 14], triacylglycerols (TAG) [15], tocopherols [15–17], and diterpenic alcohols [18–20]. Valdenebro et al. [14] found that Δ^5^avenasterol was a very adequate descriptor to establish the* arabica* percentage in roasted coffee blends, while Carrera et al. [13] proposed sitostanol in addition to Δ^5^avenasterol. González et al. [15] considered TAG and tocopherol profiles as chemical markers. Alves et al. [16] found that* arabica* coffee showed higher tocopherol contents, especially for *β*-tocopherol, while higher losses during roasting were found for *β*-tocopherol in* robusta* coffee. It was found that the ratio between *α* : *β* : *γ* tocopherol homologues might be used as a tool to distinguish the coffee type [17]. In 1994, Frega et al. [18] found some characteristic ratios among diterpenic alcohols which permitted measurement of 5–10% of the amount of* robusta* blended with* arabica* coffee. Pacetti et al. [19] proposed the ratio between kahweol and 16-*O*-methylcafestol for the authentication of Italian Espresso coffee blends. Lipid fraction was also investigated by monitoring fatty acids (FA) [21–23]. Martín et al. [21] used oleic, linolenic, linoleic, and myristic acids as chemical descriptors useful for differentiating coffee varieties by Principal Component Analysis. The same authors used also Linear Discriminant Analysis (LDA) by six FA (palmitic, stearic, oleic, linoleic, linolenic, and behenic acids) as descriptors for 100% discrimination between* arabica* and* robusta*, green and roasted, coffee samples, while in another research [22] eleven FA were useful to the same scope. Total monounsaturated fatty acids (MUFA), linolenic acid, the stearic/oleic acid ratio, and the ratio between MUFA and saturated FA (SFA) could be used to determine the relative amounts of* arabica* and* robusta* in a coffee blend [23]. More recently, spectroscopic methods have been proposed [24–26].

Accordingly, the analysis of the lipid fraction of coffee is a very interesting approach to distinguish the two varieties. In the present study, the different FA % distribution among the three* sn*- positions of TAG has been determined using a chemical-enzymatic-chromatographic procedure on roasted coffee beans of* arabica* and* robusta* varieties. In order to authenticate* arabica* roasted coffee variety, the obtained data were used as chemical descriptors in a LDA chemometric procedure with the aim of discriminating roasted* arabica* from (90 : 10)* arabica*/*robusta* mixtures.

## 2. Materials and Methods

### 2.1. Samples

A set of twenty authentic roasted coffee bean samples of different geographic origins was selected for the analysis. Fourteen samples, which belonged to the* arabica* variety (*Coffea arabica*), were from Brazil (6 samples), Colombia (4 samples), and Ecuador (4 samples). Six samples, which belonged to the* robusta* variety (*Coffea canephora*), were from Congo (2 samples), Ivory Coast (2 samples), and Uganda (2 samples). The samples were stored in a dry place in the dark at room temperature until analyses. The samples were collected in 2015 from different sellers (herbalist's shops and supermarkets). The origin and composition (100%* arabica* or 100%* robusta*) of the samples were reported on the packaging and guaranteed from the producers. The samples were tested shortly after the opening of the package and then immediately closed and left at room temperature in a dry place in the dark.

### 2.2. Reagents

All solvents and reagents were of analytical grade and were purchased from Carlo Erba Reagents (Milano, Italy).* sn*-1,2-Diacylglycerol kinase from* Escherichia coli* (DAGK; EC 2.7.1.107) was from Sigma-Aldrich (St. Louis, MO, USA). A standard mixture from Supelco (Bellefonte, PA, USA), Supelco 37 component fatty acid methyl esters (FAME) mix, containing the methyl esters of 37 FA, was used; the FA contents ranged between 2% and 4%, while the palmitic acid methyl ester was 6%.

### 2.3. Lipid Extraction

Initially, coffee bean samples were ground using a kitchen grinder (Oster, model 869-50R, USA). The extraction of the coffee lipid fraction was performed with petroleum ether using a Soxhlet apparatus, according to AOAC procedure [27]. The extract was dried over anhydrous Na_2_SO_4_ and then the solvent was evaporated using a vacuum rotary evaporator (Büchi Rotavapor B-480, Germany). Finally, the residue was weighed and dissolved in hexane.

### 2.4. Stereospecific Analysis of TAG

The TAG fraction was isolated by thin layer chromatography (TLC) from total fat of* arabica *and* robusta* samples using silica gel plates (SIL G-25, 0.25 mm, 20 cm × 20 cm, MACHEREY-NAGEL, Germany) and petroleum ether/diethyl ether/formic acid (70 : 30 : 1, v/v/v) as developing solvent, as reported in a previous paper [28]. The TAG fraction (*R*_*f*_ ≈ 0.9) was scraped off, extracted with hexane/diethyl ether (1 : 1, v/v), and subjected to transesterification to obtain the constituent fatty acid methyl esters (FAME) as described in Section 2.5.

An enzymatic procedure was carried out to obtain the FA intrapositional % composition of TAG [29]. An aliquot of TAG fraction was used to prepare the* sn*-2-monoacylglycerols (*sn*-2-MAG) by pancreatic lipase hydrolysis, according to the method provided for the Italian fat and derivate control standards [30]. The* sn*-2-MAG fraction was directly transesterified as described in Section 2.5. Another aliquot of TAG fraction was used to prepare the* sn*-1,3/*sn*-1,2(2,3)-diacylglycerols (DAG) through Grignard deacylation by adding ethyl magnesium bromide in anhydrous ethyl ether.

The mixture was shaken and then pentane (0.1% acetic acid) and water were added. The water was removed and the solution was dried over anhydrous Na_2_SO_4_ and concentrated. The* sn*-1,2(2,3)-DAG, isolated by TLC (*R*_*f*_ ≈ 0.3) using hexane/diethyl ether (1 : 1, v/v) as developing mixture, were reacted with* sn*-1,2-DAGK and Adenosine Triphosphate Disodium (Na_2_ATP) aqueous solution. The* sn*-1,2-phosphatidic acids (*sn*-1,2-PA), purified by TLC using chloroform/methanol/25% ammonia (65 : 25 : 5, v/v/v) as developing system, were transesterified as described in Section 2.5 for the following FAME analysis.

### 2.5. Preparation of Fatty Acid Methyl Esters (FAME)

The FAME of TAG,* sn*-2-MAG, and* sn*-1,2-PA fractions were prepared by transesterification as reported in Blasi et al. [28]. Hexane and 2 N methanolic KOH were added to the fraction and stirred for 3 min; after that, water was added and the upper organic phase was dried over anhydrous Na_2_SO_4_ and then analyzed by high-resolution gas chromatography (HRGC).

### 2.6. HRGC Analysis

A DANI 1000DPC gas chromatograph (Norwalk, CT, USA) equipped with a split-splitless injector and with a flame ionization detector (FID) was used [28]. The separation was obtained using the CP-Select CB for FAME fused silica capillary column (50 m × 0.25 mm i.d., 0.25 *μ*m f.t.; Varian, Superchrom, Milan, Italy). The chromatograms were acquired and processed using Clarity integration software (DataApex Ltd., Prague, Czech Republic). The injector and detector temperature was 250°C. The oven temperature was 180°C, held for 6 min and raised to 250°C at 3°C/min; the final temperature was held for 10 min. Carrier gas (He) flow rate was 1 mL/min; the injection volume was 1 *μ*L with a split ratio of 1 : 70.

A standard solution containing 37 FAME was used to identify the individual FA. The percentage of each FA was calculated using the peak area of the samples corrected with the respective correction factors, as reported by Christie [31]. The data were normalized considering only the main reported FA (% mol mean values ≥ 0.1).

### 2.7. Statistical Analysis

The results of the analyses are expressed as the mean value and standard deviation (SD) based on three replicates. Microsoft Excel 2007 (Microsoft Corporation, Redmond, WA, USA) was used for data analysis.

The data of total and intrapositional % FA compositions of TAG of (90 : 10)* arabica/robusta *mixtures were obtained using a software developed at the University of Perugia that is able to calculate their compositions from the experimental data of the stereospecific analysis of* arabica *and* robusta* pure coffee samples (each* arabica* sample was combined with each* robusta* sample and a total of 84 mixtures were obtained).

Total and intrapositional FA % compositions of TAG have been processed by Linear Discriminant Analysis (LDA) chemometric procedure with the aim of obtaining the differentiation of roasted* arabica* and (90 : 10)* arabica/robusta* mixtures. Only this proportion was chosen because the main Italian coffee processing industries use authentic 100%* arabica* or* arabica* mixed with not more than 10% of* robusta* variety. SPSS Base 10® software (Chicago, IL, USA) was used for LDA.

## 3. Results

In literature, different papers described the composition of the major TAG in coffee lipids. For example, Nikolova-Damyanova et al. [32] studied for the first time the TAG composition of crude Brazilian coffee beans and identified dipalmitolinolein, dilinoleopalmitin, and palmitoleolinolein among the main components. Jham et al. [33] used reverse-phase high-performance liquid chromatography with refractive index and light scattering detectors to determine the TAG composition of three types of coffee beans harvested in two coffee producing areas in Brazil. The methods used by the above-cited authors were called “direct analysis methods” and did not allow a complete characterization of the TAG fraction, since it was not possible to separate the molecular isomeric species and, even less, those enantiomers. The “indirect analysis methods,” based on chemical-instrumental or chemical-enzymatic-instrumental (structural or stereospecific analysis) procedures, allowed for carrying out the qualitative and quantitative analysis of all molecular TAG species, including enantiomeric ones. These procedures, called “hyphenated,” allowed the determination of the FA % compositions of each of the three* sn*- positions of TAG (% intrapositional compositions); these data could be used to obtain the interpositional distributions of FA among the three* sn*- positions of TAG [34, 35].

**Figure 1:**
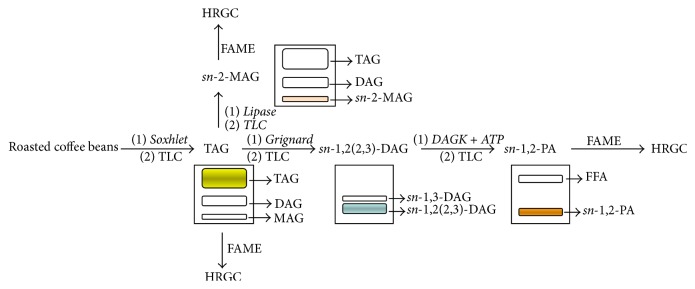
Scheme of the procedure used for TAG stereospecific analysis.

In this work, TAG stereospecific analysis was performed according to the procedure shown in Figure 1. After the initial isolation of lipid fraction by Soxhlet extraction, the TAG fraction was purified by TLC and different steps were carried out. The enzymatic hydrolysis of TAG with pancreatic lipase was used to obtain* sn*-2-MAG and, after HRGC analysis of the FAME, the acidic composition of* sn*-2- position (*A*_2_) of the glycerol backbone of TAG was obtained. TAG was also subjected to chemical hydrolysis with Grignard reagent; then, the separation of enantiomeric* sn*-1,2(2,3)-DAG, obtained by enzymatic synthesis of* sn*-1,2-PA, allowed for obtaining the acidic composition of the* sn*-1,2- positions (*A*_1,2_) of the glycerol backbone of TAG. The FA composition at the* sn*-1- and* sn*-3- positions was obtained using the % FA compositions of* sn*-1,2-PA (*A*_1,2_),* sn*-2-MAG (*A*_2_), and total TAG (*A*_*t*_), applying the following formulas:

(1) A1=2·A1,2−A2A3=3·At−A2−A1

The lipid fraction, extracted by Soxhlet with petroleum ether, represented the 11–20% of coffee bean; in the* arabica* variety, this fraction is greater than in the* robusta* one. The lipid fraction is constituted for approximately 75.0% from TAG [36].

**Figure 2:**
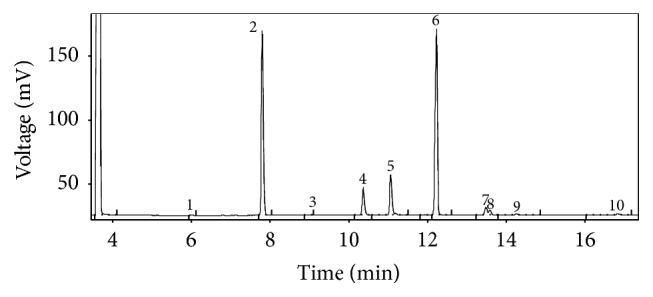
Characteristic HRGC profile of the FAME of TAG fraction of a coffee sample. (1) Myristic acid (C14:0), (2) palmitic acid (C16:0), (3) palmitoleic acid (C16:1 n-7), (4) stearic acid (C18:0), (5) oleic acid (C18:1 n-9), (6) linoleic acid (C18:2 n-6), (7) *α*-linolenic acid (C18:3 n-3), (8) arachidic acid (C20:0), (9) gadoleic acid (C20:1 n-11), and (10) behenic acid (C22:0).

The chromatogram reported in Figure 2 shows the characteristic HRGC profile of the FAME of TAG fraction. The main FA present in all samples were linoleic acid (C18:2 n-6) for unsaturated FA (UFA) and palmitic acid (C16:0) for saturated FA (SFA).

Table 1 shows the total and intrapositional FA % compositions of TAG fraction of* arabica* and* robusta* pure coffee and (90 : 10)* arabica*/*robusta* mixtures. Both coffee varieties contained high percentage of UFA (56.5–56.6%) with some significant differences relative to some FA, as oleic (C18:1 n-9) and linoleic acids.* Robusta* coffee samples showed higher content (*P* ≤ 0.05) of oleic acid than* arabica*, while on the contrary* arabica* had higher content (*P* ≤ 0.05) of linoleic acid than* robusta*. It can be observed that PUFA fraction was the most abundant in both varieties, even if* robusta* showed a higher content in monounsaturated fatty acids (MUFA) in respect to* arabica* (13.0% versus 9.1%) that had a higher content in polyunsaturated fatty acids (PUFA, 47.3% versus 43.6%). Moreover, the* arabica* coffee had the highest amount of essential FA (EFA, 47.3% versus 43.6%), represented by linoleic and *α*-linolenic (C18:3 n-3) acids. It can be observed that PUFA and EFA values were coincident. Minor FA were myristic (C14:0), palmitoleic (C16:1 n-7), gadoleic (C20:1 n-11), and behenic (C22:0), whose contents are lower than 0.6%. These data confirmed the data reported in the literature [21, 32, 36, 37].

To obtain structural information and to better characterize the TAG fraction of the considered samples, a stereospecific analysis to detect the % FA composition in the three* sn*- TAG positions was carried out. In fact, it is known that, in TAG molecules, the positions esterified by FA are numbered relative to their stereospecificity or stereospecific numbering (*sn*) as* sn*-1,* sn*-2, and* sn*-3. The type of FA and its stereospecificity in TAG molecular species largely determine the physical-chemical and nutritional characteristics of dietary fats in food products [38]. These structural elucidations are useful for characterizing the main lipid fraction of coffee and for determining the origin of the sample. Moreover, stereospecific analysis represents a powerful analytical tool useful for differentiating coffee mixtures and for detecting possible adulterations.

Table 2 shows the intrapositional FA % compositions of* arabica*,* robusta*, and (90 : 10)* arabica*/*robusta *mixtures. The stereospecific analysis data showed differences in the FA distribution; in fact, higher % value of UFA and EFA in* sn*-2- position was observed, while SFA preferred the* sn*-1- and the* sn*-3- positions. This result confirmed what is generally observable in vegetable fats; in fact, it is known that the stereospecificity of FA in TAG is characteristic for native oils and fats. Also Folstar in 1985 [39] found that UFA, especially linoleic acid, were preferably esterified with the secondary hydroxyl position in glycerol of coffee TAG. On the contrary, it was observed that TAG molecules in milk from different mammalian species largely had SFA at the* sn*-2 position and UFA at the* sn*-1(3) positions [28].

It was also observed that* arabica* coffee had higher % content of palmitic and *α*-linolenic acids in* sn*-1- position than* robusta* one, while linoleic acid was more represented inboth* sn*-1 and* sn*-3- positions. Differently, oleic acid was more represented in* robusta* coffee TAG in* sn*-1- and* sn*-3- positions.

In this study, the little differences among the considered samples highlighted from stereospecific analysis data were better revealed applying a chemometric procedure as LDA. Previous researches have shown that TAG stereospecific analysis coupled with multivariate statistical data analysis was successfully used to characterize different food products [40–42]. LDA is probably the best known and more widely used method to examine differences between groups and to discriminate them, also in the food sector. Moreover, it is known that LDA is considered an important classical parametric method for grouping samples when the sample allocation is just known. The LDA, a method of classification where the distinction between two categories is a linear function, is based on the assumption that the data obey a multivariate normal distribution and that the covariance matrix of each category (dispersion of the category) is not significantly different from one case to another.

The statistical elaborations by LDA were performed considering the following FA: palmitic, stearic, oleic, linoleic, *α*-linolenic, and arachidic acids; the variables were the total and the intrapositional FA % compositions in the three* sn*- positions (*sn*-1-,* sn*-2-, and* sn*-3-) of coffee sample TAG. The variables entered in the chemometric analysis were the ones selected by means of the multiple regression method, rejecting the variables linearity associated with the others already in the equation [43].

**Figure 3:**
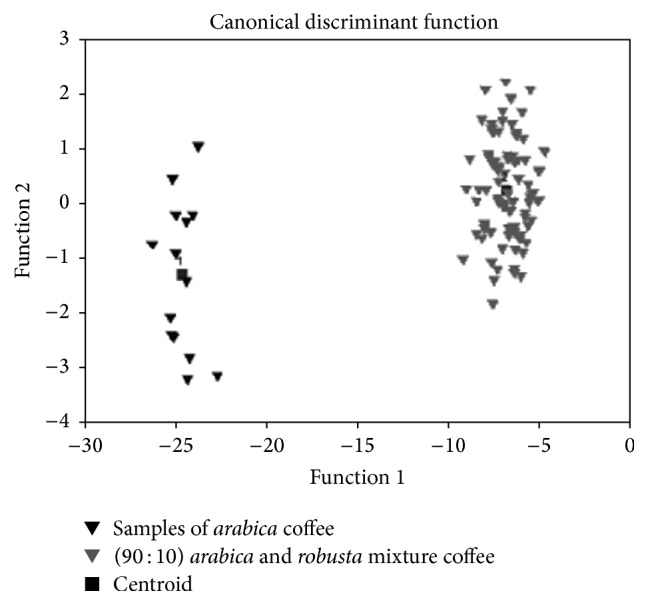
Discriminant function plot of the first two functions obtained from LDA analysis using TAG acidic compositions.

Figure 3 shows the discriminant function plot of the first two functions obtained from LDA using stereospecific analysis data. Since the main objective of the research was the setting up of an analytical tool for authentication of* arabica* coffee, in LDA statistical procedure, only pure* arabica* coffee and (90 : 10)* arabica*/*robusta* mixture data were considered. It is possible to observe that (90 : 10)* arabica*/*robusta* mixture was well discriminated from* arabica* samples. Chemometric procedure results were satisfactory and showed that this statistical approach was useful for evaluating the differences between coffee samples. The obtained results confirm that intrapositional TAG compositions, related to the specific biosynthetic pathway, represent the* fingerprint* of the analyzed matrices.

## 4. Conclusions

The stereospecific analysis represents a potent analytical-investigative procedure able to give the fingerprint of TAG fraction of each botanical variety or animal species. To the best of our knowledge, this is the first time that stereospecific analysis data of roasted* arabica* and* robusta* coffee samples have been reported. The results of this study clearly indicate that TAG stereospecific analysis data, elaborated by chemometric procedure, can be considered a valid approach for discriminating 100% authentic* arabica* coffee from (90 : 10)* arabica*/*robusta* mixture. In addition, this analytical method, based on “hyphenated” procedure, could be useful also for the geographic differentiation of coffee samples.

## Figures and Tables

**Table 1:** Total fatty acid composition (% mol, mean value ± SD) of triacylglycerol fraction of *arabica*, *robusta*, and (90 : 10) *arabica*/*robusta* mixtures.

Fatty acid	*arabica* % (*n* = 14)	*robusta* % (*n* = 6)	(90 : 10) *arabica/robusta* % (*n* = 84)
Myristic acid	0.1 ± 0.0	0.1 ± 0.0	0.1 ± 0.0
Palmitic acid	33.0 ± 1.3	32.5 ± 1.4	32.9 ± 1.1
Palmitoleic acid	0.2 ± 0.0	0.1 ± 0.0	0.2 ± 0.0
Stearic acid	7.3 ± 0.7	7.5 ± 0.3	7.4 ± 0.6
Oleic acid	8.7 ± 0.9	12.3 ± 0.7	9.0 ± 0.8
Linoleic acid	45.8 ± 1.4	42.6 ± 1.3	45.4 ± 1.2
*α*-Linolenic acid	1.5 ± 0.1	0.9 ± 0.1	1.5 ± 0.1
Arachidic acid	2.5 ± 0.2	2.9 ± 0.3	2.6 ± 0.2
Gadoleic acid	0.3 ± 0.0	0.5 ± 0.2	0.3 ± 0.0
Behenic acid	0.6 ± 0.1	0.6 ± 0.1	0.6 ± 0.1

SFA	43.5	43.4	43.4
UFA	56.5	56.6	56.6
MUFA	9.2	13.0	9.5
PUFA	47.3	43.6	47.1

SFA, saturated FA; UFA, unsaturated FA; MUFA, monounsaturated FA; PUFA, polyunsaturated FA.

**Table 2:** Fatty acid intrapositional composition (% mol, mean value ± SD) of triacylglycerol fraction of *arabica*, *robusta*, and (90 : 10) *arabica*/*robusta* mixture roasted ground coffee.

Fatty acid	*arabica*			*robusta*			(90 : 10) *arabica*/*robusta* mixture		
	*sn*-1-	*sn*-2-	*sn*-3-	*sn*-1-	*sn*-2-	*sn*-3-	*sn*-1-	*sn*-2-	*sn*-3-
Myristic acid	—	—	0.2 ± 0.0	—	—	0.3 ± 0.0	—	—	0.2 ± 0.0
Palmitic acid	51.3 ± 1.3	1.1 ± 0.0	45.3 ± 1.1	48.2 ± 1.0	1.4 ± 0.3	47.6 ± 1.0	50.3 ± 1.1	1.6 ± 0.2	45.4 ± 1.2
Palmitoleic acid	—	—	0.5 ± 0.0	—	—	0.4 ± 0.0	—	—	0.5 ± 0.0
Stearic acid	10.2 ± 0.6	0.2 ± 0.0	11.4 ± 0.6	11.2 ± 0.7	0.3 ± 0.0	10.9 ± 0.5	10.5 ± 0.8	0.3 ± 0.0	11.4 ± 0.6
Oleic acid	6.2 ± 0.8	13.1 ± 0.6	8.5 ± 0.8	10.9 ± 0.9	13.4 ± 0.7	12.6 ± 0.7	6.9 ± 0.6	12.6 ± 0.7	9.0 ± 0.9
Linoleic acid	30.6 ± 1.2	83.3 ± 2.4	23.3 ± 1.2	27.7 ± 1.4	84.0 ± 3.3	17.0 ± 1.0	30.5 ± 1.5	83.3 ± 2.3	22.6 ± 1.2
*α*-Linolenic acid	1.2 ± 0.1	2.3 ± 0.0	1.1 ± 0.1	0.5 ± 0.0	0.9 ± 0.3	1.2 ± 0.1	1.2 ± 0.2	2.1 ± 0.2	1.1 ± 0.1
Arachidic acid	0.5 ± 0.0	—	7.1 ± 0.2	1.5 ± 0.2	—	7.0 ± 0.3	0.6 ± 0.0	—	7.1 ± 0.4
Gadoleic acid	—	—	0.9 ± 0.0	—	—	1.6 ± 0.2	—	—	1.0 ± 0.0
Behenic acid	—	—	1.7 ± 0.2	—	—	1.4 ± 0.1	—	—	1.7 ± 0.1

SFA	62.0	1.3	65.7	60.9	1.7	67.2	61.4	1.9	65.8
UFA	38.0	98.7	34.3	39.1	98.3	32.8	38.6	98.1	34.2
MUFA	6.2	13.1	9.9	10.9	13.4	14.6	6.9	12.6	10.5
PUFA	31.8	85.6	24.4	28.2	84.9	18.2	31.7	85.5	23.7

## Abbreviations

DAG: Diacylglycerols
DAGK: * sn*-1,2-Diacylglycerol kinase
FA: Fatty acids
FAME: Fatty acid methyl esters
FID: Flame ionization detector
HRGC: High-resolution gas-chromatography
LDA: Linear Discriminant Analysis
MUFA: Monounsaturated fatty acids
PUFA: Polyunsaturated fatty acids
SFA: Saturated fatty acids
*sn*-2-MAG: * sn*-2-Monoacylglycerols
TAG: Triacylglycerols
TLC: Thin layer chromatography
UFA: Unsaturated fatty acids.
